# Huaier Aqueous Extract Inhibits Ovarian Cancer Cell Motility via the AKT/GSK3β/β-Catenin Pathway

**DOI:** 10.1371/journal.pone.0063731

**Published:** 2013-05-08

**Authors:** Xiaohui Yan, Tianjiao Lyu, Nan Jia, Yinhua Yu, Keqin Hua, Weiwei Feng

**Affiliations:** 1 Department of Gynecology, Obstetrics and Gynecology Hospital, Fudan University, Shanghai, China; 2 Shanghai Key Laboratory of Female Reproductive Endocrine - Related Diseases, Obstetrics and Gynecology Hospital, Fudan University, Shanghai, China; 3 Department of Experimental Therapeutics, University of Texas, M.D. Anderson Cancer Center, Houston, Texas, United States of America; Duke University Medical Center, United States of America

## Abstract

Traditional Chinese medicine has gained popularity due to its ability to kill tumor cells. Recently, the apoptotic and anti-angiogenic effects of Trametes robiniophila murr (Huaier) have been investigated. The aim of this study was to investigate its effect on cell mobility and tumor growth in ovarian cancer. Cell viability and motility were measured using SRB, scratch and migration assays. Cell apoptosis was analysed by annexin V/PI staining. Using a reverse-phase protein array (RPPA) assay, we analyzed the levels of 153 proteins and/or phosphorylations in Huaier-treated and untreated cells. Huaier inhibited cell viability and induced both early and late apoptosis in SKOV3, SKOV3.ip1 and Hey cells in a time- and dose-dependent manner. Cell invasiveness and migration were also suppressed significantly. The RPPA results showed significant differences (of at least 30%; P <0.05) in the levels of 7 molecules in SKOV3 cells and 10 in SKOV3.ip1 cells between the untreated and treated cells. Most of the molecules identified play roles in cell proliferation, apoptosis or cell adhesion/invasion. Western blot analysis further validated that Huaier treatment resulted in decreased AKT phosphorylation, enhanced expression of total GSK3β, inhibition of the phosphorylation of GSK3β on S9, reduction of both cytoplasmic β-catenin expression and nuclear β-catenin translocation, and transcriptional repression of several Wnt/β-catenin target genes (*DIXDC1, LRP6, WNT5A*, and cyclin D1). After knocking down GSK3β, β-catenin expression could not be inhibited by Huaier. Finally, Huaier inhibited the growth of ovarian tumor xenografts in vivo. These studies indicate that Huaier inhibits tumor cell mobility in ovarian cancer via the AKT/GSK3β/β-catenin signaling pathway.

## Introduction

Epithelial ovarian cancer is a leading cause of death from gynecologic cancer in the United States and the fifth most common cause of cancer mortality in women[1]. The incidence of ovarian cancer increases with age. Seventy percent of patients present with advanced disease, and less than forty percent could be cured. The overall survival of advanced ovarian cancer has not proved promising, even after surgery combined with new adjuvant strategies, such as high dose chemotherapy with peripheral blood stem cell transplantation, dose-dense weekly paclitaxel with carboplatin, and targeted therapy with carboplatin/paclitaxel. Recently, results from a randomized phase III trial (GOG 0208) showed that the use of bevacizumab (an Endothelial Growth Factor Receptor antibody) during and up to 10 months after carboplatin and paclitaxel chemotherapy prolongs the median progression-free survival by approximately 4 months in patients with advanced epithelial ovarian cancer[2]. However, the overall survival is similar to conventional therapy despite the high expense. Because none of these strategies can completely protect ovarian cancer patients from recurrence and metastasis, new drugs are urgently needed.

Among complementary therapies, traditional Chinese medicine (TCM) has gained popularity for its ability to kill tumor cells with less harm to normal cells. In addition, TCM herbal treatment is relatively inexpensive and has been reported to increase chemotherapy efficacy, reduce toxicity, prolong survival time, and improve the quality of life and immune functions[3]. Trametes robiniophila murr (Huaier) is a type of fungus from China that been used in TCM for approximately 1600 years. However, its anti-tumor effects and mechanisms have been studied only in recent years. The effective ingredients were extracted and analyzed by high-performance liquid chromatophy (HPLC) and sodium dodecyl sulfate polyacrylamide gel electrophoresis (SDS-PAGE) analyses, which identified proteoglycans as the major components of the Huaier extract, which consisted of 41.53% polysaccharides, 12.93% amino acids and 8.72% water[4], [5]. The drug, which has been approved by the Chinese Food and Drug Administration (FDA), has been used in hepatocellular cancer patients. In vitro experiments showed that Huaier can inhibit the growth of hepatocarcinoma cells (HepG2, MHCC97H), human lung adenocarcinoma cells (A549), and human breast cancer cells (MCF-7, MDA-MB-231) by inducing apoptosis[6]–[8]. Recently, Wang et al. reported that the Huaier extract might serve as a potent anti-angiogenic and antitumor agent[9]. However, whether Huaier affects the biological behavior of ovarian cells has not been explored. In this study, in addition to its anti-proliferative and apoptotic effects, the novel possibility that Huaier exerts an anti-invasive effect in ovarian cancer cells via the AKT/GSK3β/β-catenin signaling pathway was investigated.

## Materials and Methods

### Antibodies and reagents

The RPMI-1640 medium was purchased from Jinuo Co., Ltd (Shanghai, China). Fetal bovine serum (FBS) was supplied by Gibco-BRL (Rockville, IN, USA). Antibodies against AKT (1∶1000), pAKT-Thr308 (1∶1000), pS6-S235 (1∶1000), pS6-S240 (1∶1000), cyclin D1 (1∶2000), cyclin A (1∶2000),E-cadherin (1∶1000), glycogen synthase kinase 3 beta (GSK3β, 1∶1000), GSK3β phospho S9 (1∶500, Epitomics, CA, USA.) and β-catenin (1∶1000) were purchased from Cell Signaling Technology (Danvers, MA, USA). The anti-histone H1 (1∶300) and anti-mouse and anti-rabbit IgG horseradish peroxidase (HRP) (1∶5000) antibodies were obtained from Santa Cruz Biotechnology (Santa Cruz, CA, USA). Anti-GAPDH (1∶3000) and anti-β-actin (1∶3000) were purchased from Biyuntian Biotech Co., Ltd (Shanghai, China). All other chemicals were purchased from Sigma-Aldrich (Saint Louis, MO, USA) and Biyuntian Biotech Co., Ltd.

### Preparation of Huaier aqueous extract

The electuary ointment of Huaier was a gift from Gaitianli Medicine Co. Ltd (Jiangsu, China). The preparation methods for the Huaier extract and its ingredients have been described elsewhere [4], [5] A total of 2 g of the electuary ointment was dissolved in 50 ml of complete medium and sterilized with a 0.22 µm filter to obtain the 40 mg/ml stock solution, which was stored at −20°C.

### Cell culture

The three types of ovarian epithelial cancer cell lines were used in this study. SKOV3 and Hey cells were obtained from the American Type Culture Collection. SKOV3.ip1 cells were gifts from the University of Texas MD Anderson Cancer Center (Houston, TX)[10]. Cell lines were routinely maintained in RPMI-1640 medium containing 10% FBS, 100 U/ml penicillin and 100 µg/ml streptomycin. The cells were incubated at 37°C in 5% CO_2_.

### Cell proliferation assay

The growth inhibitory effect of Huaier was determined using a sulforhodamine B (SRB) assay. Briefly, SKOV3 cells (2.5×10^3^),SKOV3.ip1 cells (2×10^3^) and Hey cells(1.5×10^3^) were seeded in 96-well plates and treated with the Huaier aqueous extract at different concentrations. At the indicated times, the cells were washed, fixed with 30% (wt/vol) trichloroacetic acid, and stained for 30 minutes at room temperature with 0.4% sulforhodamine B (SRB) in 1% acetic acid. The dye was washed off with 1% (vol/vol) acetic acid and then dissolved in a 10 mM Tris base solution. The plates were read with a microplate reader (Bio-Rad) at 570 nm. Each experiment was conducted in triplicate and repeated at least three times.

### Annexin V/PI staining

The Dead Cell Apoptosis Kit with Annexin V Alexa Fluor® 488 & Propidium Iodide (PI) (Invitrogen, CA, USA) was used to investigate whether treatment with Huaier induced the SKOV3 cells, SKOV3.ip1 cells and Hey cells to undergo apoptosis. The cells were fluorescently labeled following the instructions from the manufacturer. In brief, cultured SKOV3 cells (2×10^5^), SKOV3.ip1 (2×10^5^) cells and Hey cells (5×10^3^) in a 35 mm dish were treated with different concentrations of Huaier for 48 hours. The cells were harvested and resuspended at 1 × 10^6^ cells/ml in binding buffer. Then, green fluorescent dye (AlexaFluor® 488)-conjugated annexin V and PI were added and incubated in the dark for 15 min at room temperature. The samples were analyzed using a FACScan flow cytometer (Beckman) equipped with CellQuest software. The percentage of apoptotic cells was calculated from the percentage of cells in the lower right (LR) quadrant of the dot plot, which represents the number of early apoptotic cells (annexin V+/PI−) divided by the total number of cells. Each experiment was repeated at least three times.

### In vitro scratch assay

An in vitro scratch assay was performed to assess how cell migration was affected by administration of the Huaier aqueous extract. A total of 2×10^4^ cells were seeded into a 24-well plate. Reference points near the “scratch” were marked to ensure the use of the same area for the image acquisition. After a 24-hour incubation period, confluent monolayers of the SKOV3, SKOV3.ip1 and Hey cells were scratched using a 200 µl pipette tip to create a straight “scratch”. After washing 2 times with PBS, serum-free medium containing Huaier extract (5 mg/ml) was added to each well. The scratch width was measured at four predefined locations at the start and at 12 and 24 hours after the scratch in SKOV3 and SKOV3.ip1 cells. And the scratch width was measured in Hey cells at the start and at 6 and 12 hours after scratch. The distances between the 2 edges of the scratch were measured at the reference points and analyzed statistically.

### Matrigel invasion assays

The Transwell system (24-well insert; pore size, 8 µm; Corning Costar, Lowell, MA, USA) was used to explore the effect of the Huaier aqueous extract on the invasiveness of the SKOV3, SKOV3.ip1 and Heycells. The inserts were coated with 30 µl of Matrigel (B.D. Biosciences Pharmingen). A total of 3×10^4^ SKOV3, SKOV3.ip1 and Hey cells each suspended in 0.2 ml of fresh medium without fetal bovine serum were added to the upper well of the chamber. In the control group, 600 µl of complete medium was added to the lower well, whereas in the test group, the medium contained 5 mg/ml Huaier. After incubation for 24 hours, the cells on the upper surface of the membrane were swiped with cotton swabs. Then, the cells adhering to the lower surface of the inserts were fixed and stained with hematoxylin. Six representative fields of each insert were randomly counted using an Olympus light microscope.

### Reverse-phase protein array

SKOV3 cells and SKOV3.ip1 cells (5× 10^5^) were cultured in 6-cm diameter dishes. After a 24 hour incubation, the cells were divided into four groups to receive different treatments: complete medium only for 72 hours, Huaier aqueous extract (5 mg/ml) for 72 hours, complete medium for 60 hours plus overnight starvation and 10 min stimulation with epidermal growth factor (EGF, 20 ng/ml) and Huaier aqueous extract (5 mg/ml) for 60 hours plus overnight starvation and 10 minutes stimulation with EGF (20 ng/ml). The reverse-phase protein array (RPPA) assay was performed at the Functional Proteomics Reverse Phase Protein Array Core Facility at the M.D. Anderson Cancer Center. Briefly, the cell lysates were adjusted for their protein concentrations, denatured with SDS, and serially diluted (from undiluted to 1∶16 dilution) to define the linear range of each antigen-antibody reaction. The lysates were spotted onto nitrocellulose-coated slides (Whatman, Inc.) using a GeneTAC G3 microarrayer (Genomic Solutions) along with positive and negative controls. A total of 153 validated antibodies specific for proteins or their phosphorylated sites that are involved in various signaling pathways were available and used in the RPPA (See Table S1 for proteins and phosphorylation sites used in RPPA studies). Each slide was probed with a primary antibody plus a conjugated secondary antibody using the Dako CSA (tyramide) amplification approach and visualized using the DAB colorimetric reaction. The data collected were quantified using the quantitative software MicroVigene, which was specifically developed for this approach. The protein phosphorylation levels were expressed as a ratio to the equivalent total proteins. The values derived from the slope and the intercepts were expressed relative to the standard control cell lysates on the array. All values were compared with the mean within each antibody probe and visualized with heatmaps created by the software MatLab (Mathworks Inc.).

### Cell fractionation

Nuclear proteins were isolated using the BestBio Nuclear and Cytoplasmic Extraction reagents (BestBio, Shanghai, China) according to the manufacturer's specifications. Briefly, 1×10^6^ cells were washed twice with cold PBS and lysed with 100 µl ice-cold cytoplasmic extraction reagent (CER) and 1 µl protease inhibitor cocktail. After collecting the cytoplasmic fraction (supernatant), the nuclear extraction reagent (NER) and protease inhibitor cocktail were added to the insoluble fraction, maintaining the volume ratio of CER:NER:protease cocktail at 100∶500∶1. Following a 60 min incubation and a 5-min centrifugation (16,000 × *g* at 4°C), the nuclear fraction was collected. The supernatant and nuclear fraction were subjected to western blot analysis for β-catenin.

### Western blot analysis

The cells were plated at a density of 2×10^5^ per 3.5-cm diameter dish and collected after the indicated treatment. The cells were lysed in lysis buffer (Biyuntian Biotech Co., Ltd, Shanghai, China) following the instructions from the manufacturer. Equal amounts of protein (20 µg per lane) were separated by 12% SDS-PAGE and transferred to PVDF membranes (Millipore, Billerica, MA). After blocking, the blots were probed with the primary antibodies and incubated overnight at 4°C, followed by labeling with the appropriate secondary antibodies conjugated with HRP. Immuno-reactive bands were visualized using the ECL detection system (Pierce, Rockford, IL, USA). GAPDH, β-actin and histone H1 were used as the loading controls.

### Immunocytochemistry

Cultured cells were washed with PBS and fixed with 4% paraformaldehyde. The slides were washed again, treated with 1% Triton for 15 minutes and blocked with 3% H_2_O_2_ for 20 min. After washing, the slides were blocked with normal goat serum for 10 minutes at RT and incubated first with 1∶200 anti-human E-cadherin antibody (Epitomics, CA, USA) at 4°C overnight and then with a biotinylated anti-rabbit secondary antibody for 30 minutes at RT. Then, the slides were incubated with the avidin-biotin-peroxidase system for 10 minutes at RT and stained with diaminobenzidine (DAB) for 2 minutes. Lastly, they were counterstained with hematoxylin and viewed under a light microscope.

### Quantitative real time RT-PCR

The cells were treated with or without 7.5 mg/ml Huaier for 48 h. Total RNA was extracted from the treated and control cells using TRIzol reagent (Invitrogen) according to the manufacturer's instructions. cDNA was synthesized from 1 µg of RNA using the RevertAid First Strand cDNA Synthesis kit (Fermentas, St-Leon-Rot, Germany). The expression levels of genes related to the Wnt signaling pathway [DIX domain containing-1(*DIXDC1)*], low density lipoprotein receptor-related protein 6(*LRP6)*, and wingless-type MMTV integration site family, member 5A(*WNT5A)*) were evaluated with an Illumina Eco Real-time system using a Perfect Real Time Kit (Takara). β-actin was used as an internal control in each reaction. The primer sets for these genes will be supplied upon request. The relative fold changes were calculated using the ΔΔCt method.

### Mouse xenograft and measurement of tumor size

The animal experiments were approved by the ethical committee of the Obstetrics and Gynecology Hospital of Fudan University. Six-week-old female BALB/c mice were purchased from the Sino-British SIPPR/BK Lab Animal Ltd., Co (Shanghai, China). SKOV3 cells (4 × 10^6^) were injected subcutaneously into the right flank of each mouse. Then, the mice were randomly divided into four groups (n = 6 mice/group) to receive either normal saline (NS) as a control, Huaier (4 g/kg body weight), cisplatin (DDP) (5 mg/kg bodyweight) or Huaier plus DDP. Huaier was administered orally daily. DDP was injected once a week for three weeks. The control mice were treated with the same volume of NS only. Tumor volumes were measured twice a week using a digital caliper and calculated using the following formula: tumor volume (mm^3^)  =  (tumor length [mm] ×tumor width× height [mm]) π/6. The mice were killed at day 42 after tumor cell injection. Body weights were also measured to assess the side effects. The means of three independent measurements were averaged.

### siRNA-mediated Gene Silencing

The GSK3β siRNA duplexes which target the sequences: (5′-GAGCAAAUCAGAGAAAUGAdtdt-3′) was synthesized by Genechem (Shanghai, China), For siRNA transfection, 5 × 10^5^ cells/dish were plated in 60 mm culture dishes. The following day, 10 µl of Lipofectamine 2000 reagent (Invitrogen, Carlsbad, CA) was added to 0.5 ml Opti-MEM reduced serum medium(Invitrogen, Carlsbad, CA) without antibiotics and serum, and incubated at room temperature for 5 min (solution A). 200 pmol siRNA was added to 0.5 ml Opti-MEM medium without antibiotics and serum (solution B). Solution A and solution B were then mixed and incubated at room temperature for 20 min. The cell culture medium was removed, and then 1 ml of the Lipofectamine 2000-siRNA mixture and 4 ml of fresh 1640 medium without antibiotics were added to each culture dish and gently mixed. 24 hours later, the medium was replaced with fresh culture medium or 5 mg/ml Huaier aqueous extract. Cells were harvested for western blot analysis after 24 hours of incubation.

### Statistical analysis

The data are presented as means ± standard deviation. Experiments were repeated three times. The results were analyzed using the SPSS 11.5 software. p<0.05 was accepted as significant.

## Results

### Huaier inhibited cell viability in SKOV3, SKOV3.ip1 and Hey cells

**Figure 1:**
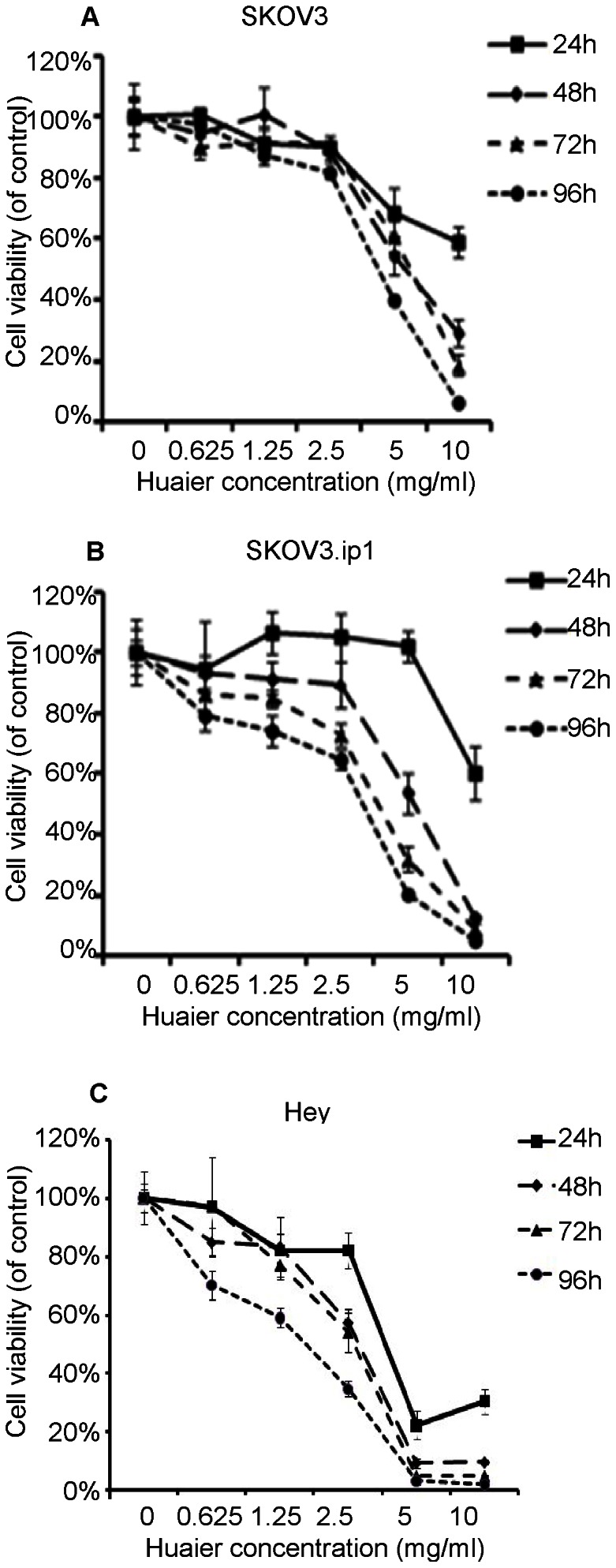
Huaier extract inhibits ovarian cancer cell proliferation in vitro. The growth inhibitory effect of Huaier was measured using the SRB assay. SKOV3 (A)SKOV3.ip1 (B) and Hey (C) cells were treated with Huaier for 24, 48, 72 and 96 h. The experiments were performed in triplicate, and the data are presented as the means±SD of three separate experiments.

To evaluate the effect of the Huaier extract on the SKOV3, SKOV3.ip1 and Hey cells, we measured cell viability using the SRB assay. After the cells were treated with Huaier for 24, 48, 72 and 96 hours at various concentrations (0, 0.625, 1.25, 2.5, 5 and 10 mg/ml), Huaier significantly inhibited the growth of the SKOV3, SKOV3.ip1 and Hey cells in a time- and dose-dependent manner (Figure 1). The cytotoxic effect started 24 hours after the 5 mg/ml Huaier treatment and became more evident at 48, 72 and 96 hours in the SKOV3 and Hey cells, whereas it started at 48 hours in the SKOV3.ip1 cells. The Hey and SKOV3.ip1 cells were more sensitive to the Huaier treatment, as the viability rates were decreased to 31.94% (72 hours, p<0.001) and 20.43% (96 hours, p<0.001) in the SKOV3.ip1 cells, 4.9% (72 hours, p<0.001) and 3.1% (96 hours, p<0.001) in the Hey cells compared with 61.1% (72 hours, p = 0.01) and 40.1% (96 hours, p<0.001) in the SKOV3 cells. A significant decrease in cell viability was observed when the cells were treated with 10 mg/ml Huaier, independent of treatment duration.

### Cell apoptosis analyzed using PI-annexin-V staining

**Figure 2:**
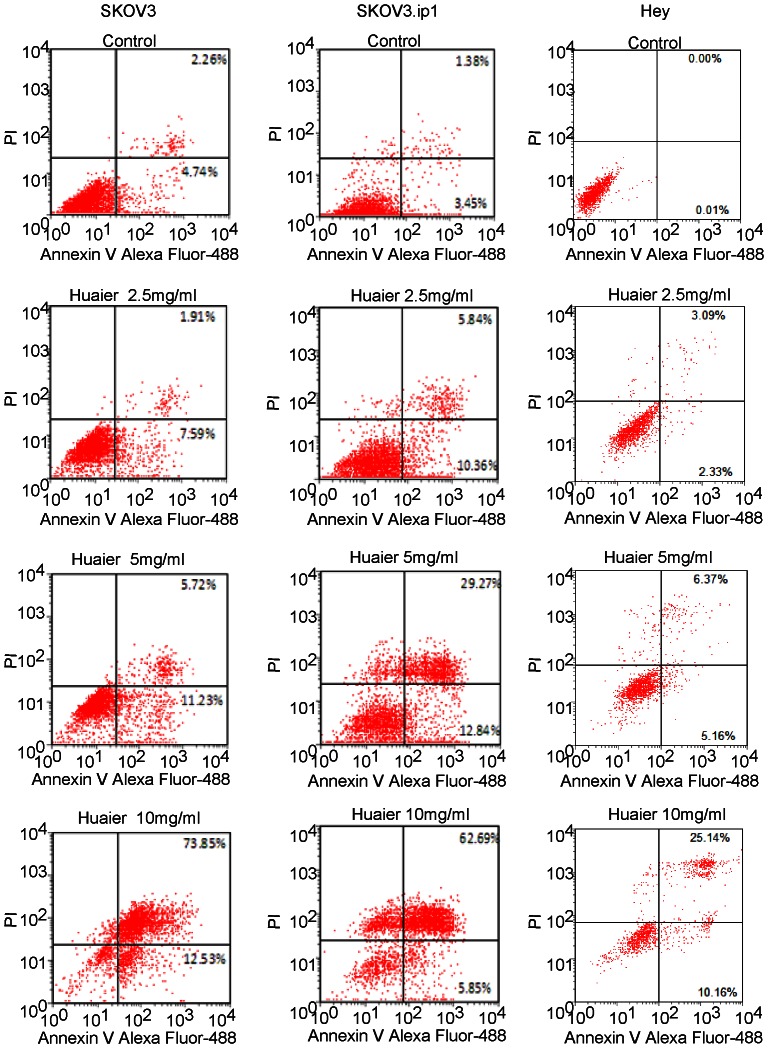
Huaier induces apoptosis in ovarian cancer cells in vitro. The percentage of apoptotic cells were measured by flow cytometry using the PI-annexin V assay. (A, B,C) Dot plots showing apoptosis in SKOV3 (A), SKOV3.ip1 (B) and Hey (C) cells with Huaier treatment at 0, 2.5, 5 and 10 mg/mL for 48 h.

Because the Huaier extract significantly inhibited cell growth, we further explored whether this effect was achieved by inducing apoptosis. The PI-annexin V staining assay showed that after Huaier treatment for 48 h, the late apoptosis or cell death rate (UR) and the early apoptosis rate (LR) both increased in a dose-dependent manner in SKOV3, SKOV3.ip1 and Hey cells (Figure 2 and Table 1).

**Table 1:** Percentage of quadrant distribution (QD) in the PI-annexin-V staining apoptosis assay.

QD	48 h-control	48 h-2.5 mg/ml	48 h-5 mg/ml	48 h-10 mg/ml
SKOV-3 cell line				
UL	0.06±0.01	0.08±0.03	0.37±0.06	4.55±0.83
UR	1.94±0.08	1.82±0.15	5.13±0.39**	65.92±1.2**
LL	92.51±0.24	90.64±0.55	82.04±0.81	16.88 ±0.92
LR	5.50±0.21	7.47±0.46**	12.46±0.5**	12.6±0.57**
SKOV-3-ip cell line				
UL	0.34±0.26	0.78±0.05	8.63±1.64	25.59±1.02
UR	1.3±0.38	6.24±0.76**	33.73±4.58**	56.0±2.49**
LL	95.08±0.93	84.67±1.41	45.48±2.1	12.58 ±1.13
LR	3.27±0.82	8.32±0.62**	12.16±1.13**	5.78 ±0.56*
Hey cell line				
UL	0.02±0.19	0.92±0.21	3.75±0.21	2.62±0.14
UR	0.00±0.02	3.09±0.16**	6.37±1.37**	25.14±0.83**
LL	99.97±1.22	93.66±1.35	84.73±1.66	62.08±2.96
LR	0.01±0.01	2.33±1.15	5.16±0.73**	10.16±1.54**

The data presented are the mean ± SD of three independent experiments. LL, Lower Left; LR, Lower Right; UL, Upper Left; UR, Upper Right *: P<0.05, **: P<0.01.

UR (upper right) indicates the percentage of cells undergoing late apoptosis, i.e., the cell death rate. LR (lower right) indicates the percentage of cells undergoing early apoptosis. * p<0.05 **P<0.01 compared with the control.

### Cell motility decreased due to exposure to Huaier extract

**Figure 3:**
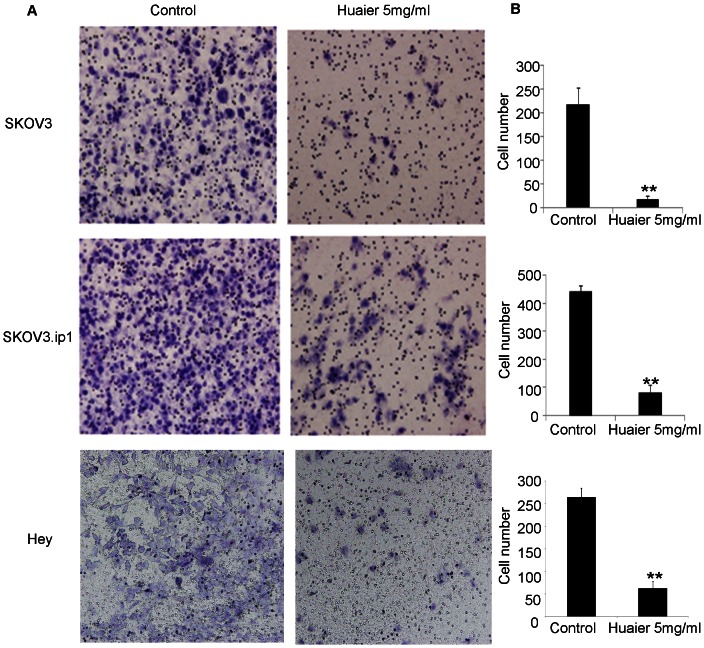
Huaier inhibits the invasion of ovarian cancer cells in vitro. (A) The effect of Huaier on the invasion of SKOV3, SKOV3.ip1 and Hey cells was examined using the Transwell system. Representative images are presented. (B) After a 24 hour treatment with 5 mg/ml Huaier, the number of successfully invading SKOV3, SKOV3.ip1 and Hey cells was counted. The data are presented as means ± SD. ** P<0.01 compared with the control.

To explore whether the Huaier extract affected cell motility, cell invasion and scratch assays were performed. When the SKOV3 cells were treated with 5 mg/ml Huaier extract, the number of invading cells through the Matrigel-coated membrane was significantly decreased compared with the untreated group (218±35 versus 18±7, p<0.001,), indicating that invasion ability was inhibited. Similar results were found in the SKOV3.ip1 and Hey cells (SKOV3.ip1:438±26 versus 83±25; Hey: 264±21 versus 62±16 p<0.001, Figure 3).

**Figure 4:**
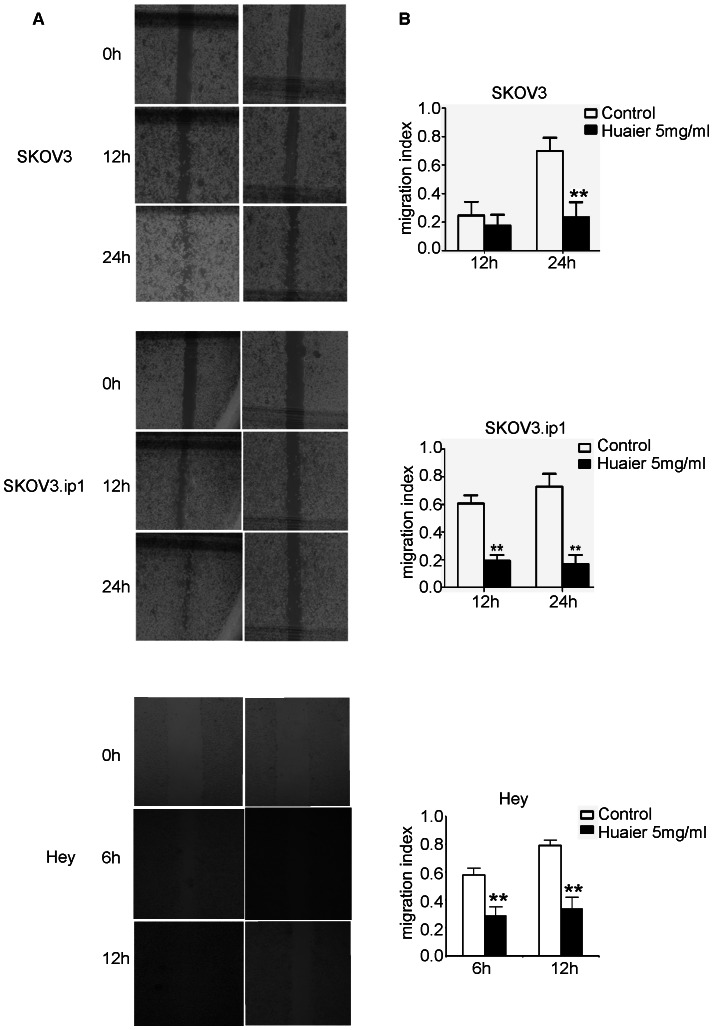
Huaier inhibited the migration of ovarian cancer cells in vitro, as measured in a scratch assay. (A) A straight wound area was generated in each culture well at the beginning, and administration of Huaier (5 mg/ml) showed a delayed process of wound closure compared with the untreated cells. Representative images of the control cells and 5 mg/mL Huaier-treated cells were acquired at 0, 12,24 h (SKOV3, SKOV3.ip1) or 0, 6, 12 h (Hey). (B) The migration indices were significantly inhibited by Huaier. The bars represent the migration index for each treatment, expressed as a value relative to the distance moved by the cell monolayer. The data are presented as means ± SD. The experiments were repeated at least three times. ** p<0.01 compared with the control.

A scratch assay was performed to assess the cell migration in vitro. As indicated in Figure 4, the migration index (corresponding to wound healing capacity) was significantly inhibited in SKOV3 cells treated with Huaier for 48 hours compared with untreated cells (23.3% versus 69.8%, p<0.01), whereas it was similar in cells treated with Huaier for 24 hours and untreated cells (17.5% versus 24.7%, p>0.05). However, the migration index decreased as early as 24 hours after the Huaier treatment in SKOV3.ip1 cells compared with untreated cells (19.3% versus 60.6%, p<0.05). For Hey cells, the wound can almost closed up for only 12 hours healing in untreated cells, and the migration index was significantly inhibited in treated cells with Huaier for merely 6 h (58.2% versus 29.1%. p<0.01).

### Cell proliferation, apoptosis and cell adhesion or invasion signaling proteins are down-regulated by Huaier treatment

To investigate the signaling pathways that might contribute to Huaier's inhibitory effect on motility, we used RPPA analysis to assess protein changes in SKOV3 and SKOV3.ip1 cells treated with Huaier. RPPA is a new high-throughput technology that monitors changes in protein expression over time, before and after treatments, between disease and non-disease states and between responders and non-responders[11]. Using RPPA, we analyzed the expression level of 153 proteins and/or phosphorylation sites in Huaier-treated or untreated ovarian cancer cell lines with or without EGF stimulation. For each sample and each antibody, the signal difference between the untreated and Huaier-treated cells was calculated as follows[12]: ([mean of treated cells - means of non-treated cells]/[means of non-treated cells]) ×100%.

**Figure 5:**
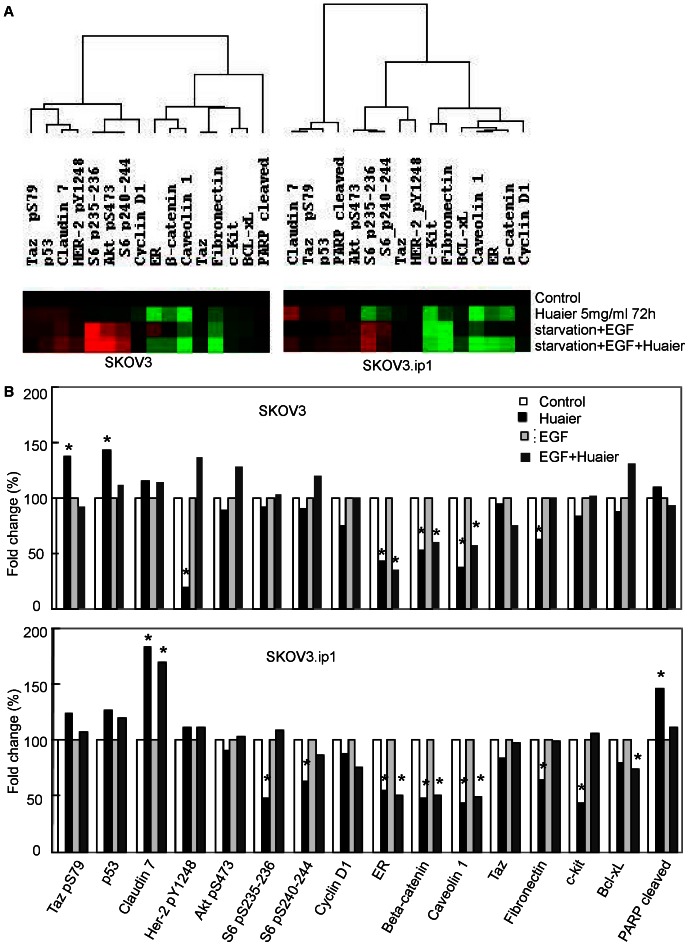
Huaier regulates proteins related to cell proliferation, apoptosis and cell adhesion or invasion in ovarian cancer cells, as detected by the reverse phase protein array (RPPA) assay. (A) Hierarchical cluster analysis of four samples (control, Huaier-treated for 72 hours, starvation+EGF stimulation, starvation +EGF stimulation+Huaier for 60 hours) in SKOV3 and SKOV3.ip1 cells according to the expression of 16 signaling molecules. Cluster color key: red – up-regulated; green – down-regulated; black – unchanged. (B and C): Fold changes in the expression or phosphorylation of various proteins after Huaier treatment and addition of EGF were analyzed using the RPPA assay in the SKOV3 (B) and SKOV3.ip1 (C) cells. The relative values of the control group and the starvation+EGF stimulation groups were set as 1.0. *: fold change>30%.

Of the 153 proteins and phosphorylation sites analyzed, 7 and 10 differed by more than 30% in the treated and untreated conditions in the SKOV3 and SKOV3.ip1 cells, respectively. Representative heatmaps are presented in Figure 5A. In SKOV3 cells, Huaier treatment inhibited cell proliferation and altered the levels of related proteins/phosphorylation sites; for example, it decreased HER2 phosphorylation at Y1248 and ER protein levels but up-regulated p53 and Taz phosphorylation at S79. In SKOV3.ip1 cells, more pro-proliferative proteins, such as ER, c-kit and phosphorylated S6 (ribosomal protein S6 kinase; phosphorylated at S235-236 and S240-244) were down-regulated by Huaier. In addition, Huaier inhibited the expression of the longer isoform of Bcl (Bcl-xL) and up-regulated the expression of pro-apoptotic proteins, such as p53 and cleaved poly(ADP-ribose) polymerase (PARP_cleaved), in SKOV3.ip1 cells (Figure 5B).

We also found that Huaier may regulate cell adhesion and invasion, based on down-regulation of fibronectin, β-catenin and caveolin-1 expression in both cell lines and up-regulation of claudin 7 in SKOV3.ip1 cells after Huaier treatment (Figure 5B).

### Involvement of the pAKT/mTOR/S6 pathway in Huaier-induced ovarian cancer cell growth inhibition

**Figure 6:**
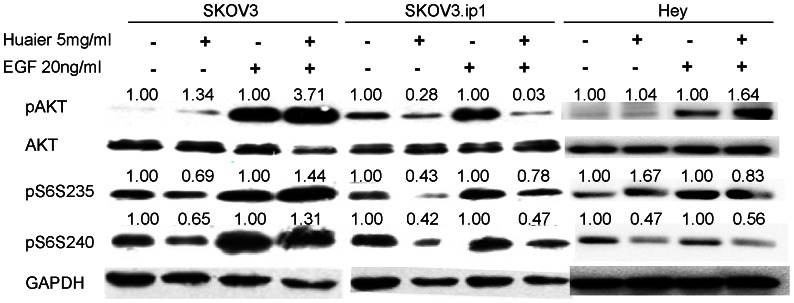
Huaier alters AKT and S6 kinase expression in SKOV3, SKOV3.ip1 and Hey cells. AKT phosphorylation, total AKT levels, and S6 phosphorylation at S235 and S240 were measured by western blotting of samples from each of the four treatment groups (control, Huaier-treated for 72 hours, starvation+EGF stimulation, starvation+EGF stimulation+Huaier for 60 hours). The numbers on the protein bands represent the fold changes, with the basal levels in the control or starvation +EGF groups assigned a value of 1.0.

Using the RPPA, we found that ribosomal S6 kinase phosphorylation was repressed by Huaier treatment. S6 kinase is a major target of the mTOR pathway, and it promotes cell growth and proliferation. The AKT pathway is an upstream activator of the mTOR pathway. Therefore, we analyzed the protein levels of pAKT, AKT, pS6 (S235–236) and pS6 (S240–244) by western blotting. We found that AKT phosphorylation was significantly reduced in SKOV3.ip1 cells after 72 h of Huaier treatment, either with or without EGF stimulation. In accordance with the decrease in pAKT, the phosphorylation of S6 at S235–236 and S240–244 was also down-regulated. However, in SKOV3 cells, the phosphorylation of S6 at S235–236 and S240–244 was down-regulated in cell treated with Huaier only but not in the EGF stimulation groups, whereas pAKT was slightly increased in the Huaier-treated SKOV3 cells. Huaier down-regulated the phophorylation of S6 at S240–244 in both Huaier-treated only and EGF stimulation groups in Hey cells. (Figure 6).

### Huaier inhibits cellular invasion via the GSK3β/β-catenin pathway

Because the results of the annexin V/PI staining indicated that Huaier induces apoptosis and the RPPA results further indicated that several pro- and anti-apoptotic proteins were modulated by Huaier treatment, our results support the pro-apoptotic effect of Huaier observed in other studies. However, the RPPA analysis also showed that proteins involved in cell adhesion and invasion were modulated by Huaier treatment (Figure 5). This suggests that Huaier may have another therapeutically important effect, especially because epithelial ovarian cancer cells have a unique invasive/metastatic capacity and often implant into the pelvic or abdominal cavity.

**Figure 7:**
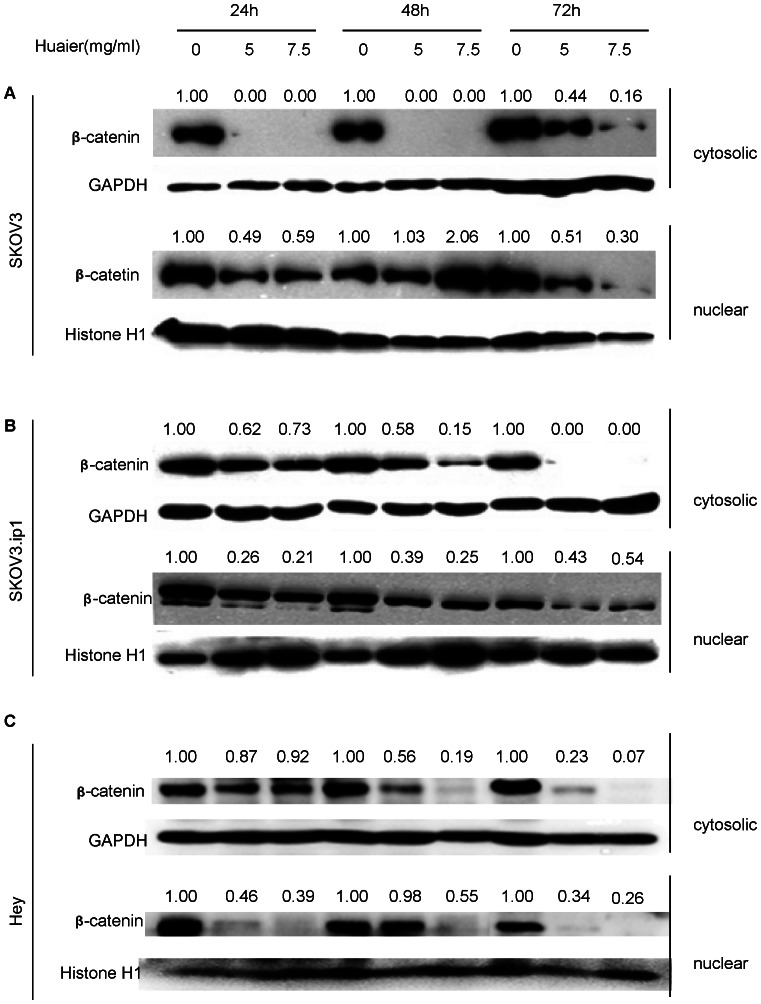
Huaier alters β-catenin dynamics. Both the cytoplasmic expression and nuclear translocation of β-catenin were significantly inhibited upon Huaier treatment. (A) SKOV3, (B) SKOV3.ip1 and (C) Hey cells were treated with 5 mg/ml and 7.5 mg/ml Huaier for 24, 48 and 72 h. The cytoplasmic and nuclear proteins were extracted separately. A representative western blot is shown. For densitometric analysis of β-catenin levels, the results were normalized to the levels of GAPDH (for cytoplasmic proteins) or histone 1 (nuclear proteins). The basal level for the untreated groups at each time point was assigned a value of 1.0.

Among those proteins, β-catenin is a key component of the E-cadherin-mediated cell–cell adhesion cascade. Using western blots, we further verified the changes in β-catenin expression in SKOV3, SKOV3ip1 and Hey cells. As shown in Figure 7, Huaier dramatically decreased the cytosolic accumulation of β-catenin in a time-dependent manner in SKOV3, SKOV3.ip1 and Hey cells. The nuclear expression of β-catenin was also decreased. Our data indicate that Huaier represses not only the protein expression, but also the nuclear translocation of β-catenin.

**Figure 8:**
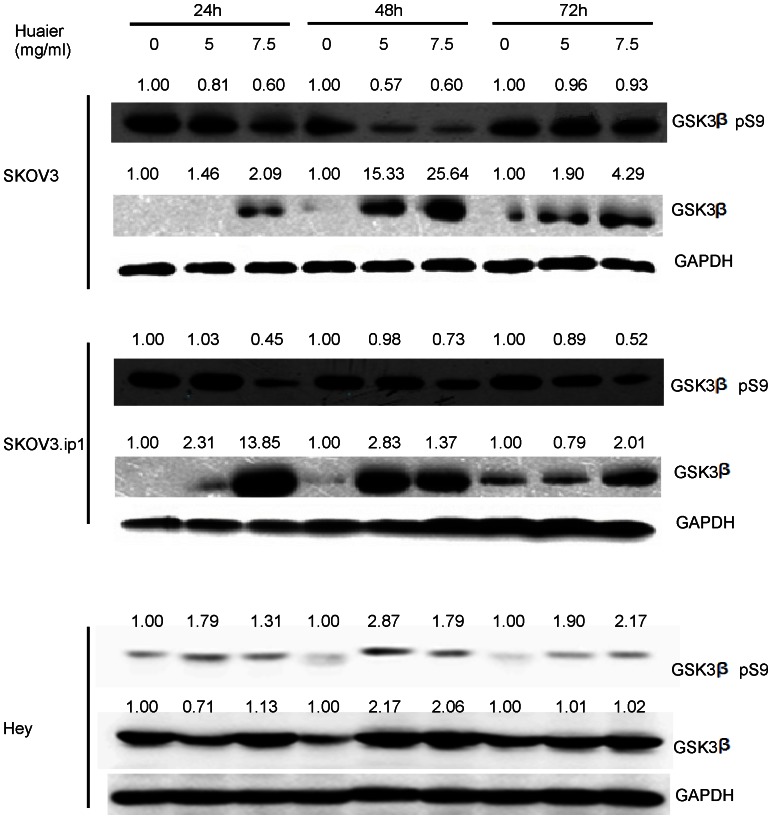
Huaier treatment enhances total GSK3β expression and inhibits phosphorylation of GSK3β at Ser 9 in SKOV3, SKOV3.ip1 and Hey cells. (A) SKOV3, (B) SKOV3.ip1 and (C) Hey cells were treated with 5 mg/ml and 7.5 mg/ml Huaier for 24, 48 and 72 h. A representative western blot is shown. The basal level for the untreated groups at each time point was assigned a value of 1.0.

To clarify the mechanism causing the down-regulation of β-catenin, we further evaluated the expression of GSK3β (glycogen synthase kinase 3β), which is known to negatively regulate the classic Wnt/β-catenin signaling pathway by phosphorylating β-catenin, which leads to its degradation[13]. Because phosphorylation of GSK3β on S9 inhibits its activity and prevents the targeting of β-catenin for degradation, whole cell lysates were examined for both total GSK3β and phosphorylated GSK3β at S9 levels following Huaier treatment. As shown in Figure 8, the Huaier treatment enhanced total GSK3β expression dramatically (13.85∼25.64 fold) in a dose-dependent manner in both cell lines. In the SKOV3 cells, the highest expression of total GSK3β was observed at 48 hours after Huaier treatment, whereas in the SKOV3.ip1 and Hey cells, the highest expression was observed at 24 hours. In addition, GSK3β S9 phosphorylation was inhibited by Huaier treatment in both cell lines. Thus, the levels of activated GSK3β were dramatically enhanced by the Huaier treatment.

In normal epithelial cells, β-catenin links the cytoplasmic tails of E-cadherin molecules, which form adherens junctions with the neighboring cells. Because the loss of E-cadherin expression enables carcinoma cells to become invasive, we measured E-cadherin expression after Huaier treatment in the three cell lines. As expected, the E-cadherin expression was increased in Huaier-treated Hey cells. However, it was not changed in the SKOV3 and SKOV3.ip1 cells, as shown by western blot analysis (Figure S1).

### Huaier cannot repress β-catenin in the GSK3β-silenced ovarian cancer cells

**Figure 9:**
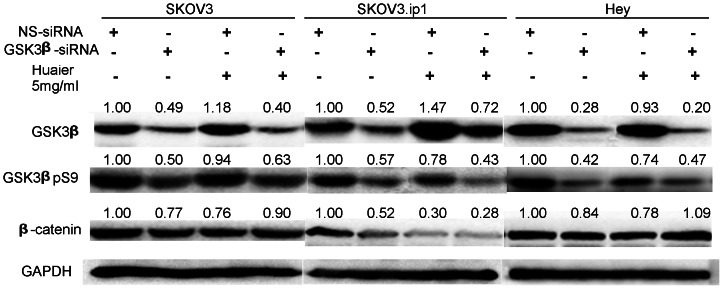
Huaier could not inhibit β-catenin in SKOV3, SKOV3.ip1 and Hey cells after specific GSK3β siRNA transfection. Cells were transfected with non-specific siRNA (lane 1, 5 and 9) and with GSK3β specific siRNA only (lane 2,6 and 10). Cells were transfected with non-specific siRNA (lane 3,7and 11) or GSK3β specific siRNA (lane 4, 8 and 12) for 24 hours and then treated with 5 mg/ml Huaier for additional 24 hours. Western blot results show GSK3β, phosphor-GSK3β pS9 and β-catenin expression. The basal level for the non-specific siRNA groups was assigned a value of 1.

Compared to non specific siRNA groups, total GSK3β and phosphor-GSK3β at S9 were both successfully decreased by GSK3β specific siRNA in SKOV3, SKOV3.ip1 and Hey cells without Huaier treatment. Accordingly, β-catenin was not dramatically changed in three cell lines. (Figure 9, lane 1,2, 5,6, 9,10). In addition, non-specific siRNA didn't interfere with Huaier's up-regulation of GSK3β and down-regulation of β-catenin. (Figure 9, lane 1,3,5,7,9,11). However, after knocking down GSK3β, Huaier treatment couldn't significantly inhibit β-catenin expression. Even in Hey cells, the expression of β-catenin was slightly increased. (Figure 9, lane 2,4,6,8,10,12).

### Huaier treatment represses the expression of Wnt/β-Catenin target genes

**Figure 10:**
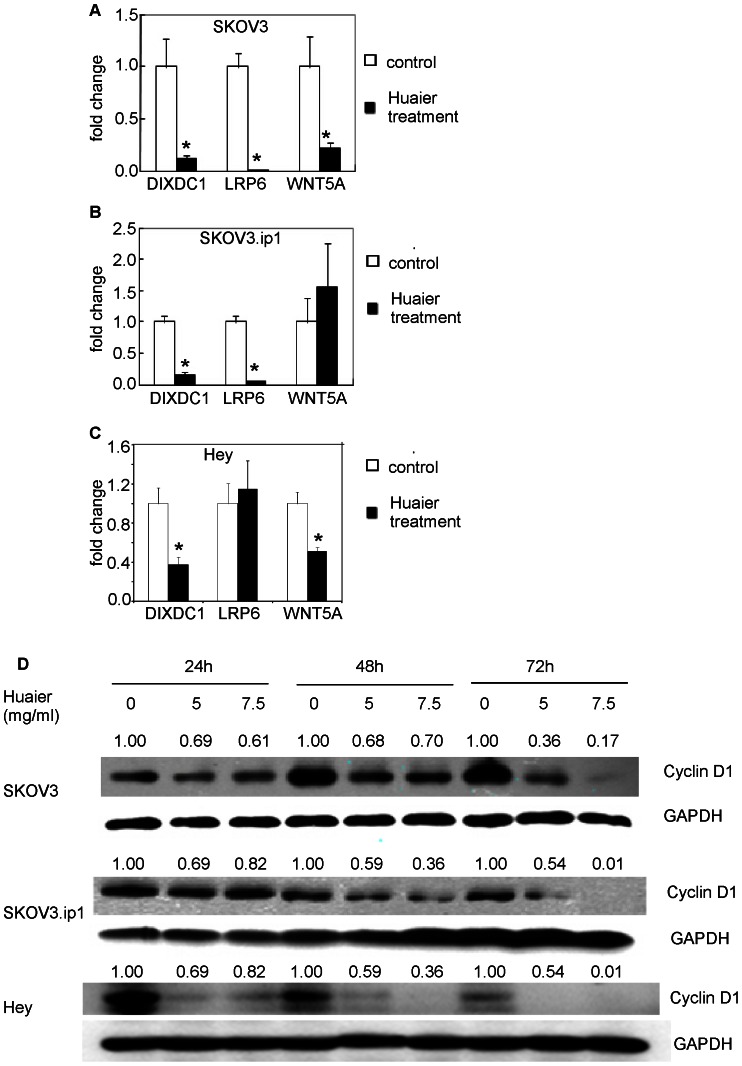
Huaier treatment represses the expression of Wnt/β-catenin target genes. (A) Huaier treatment altered the transcription of DIDX2, LRP6 and Wnt 5A genes in the SKOV3, SKOV3.ip1 and Hey cells. The gene expression was measured by real-time PCR in cells treated with 7.5 mg/ml Huaier for 48 h, and untreated cells served as the control. (B) Cyclin D1 protein expression was suppressed after treatment with 5 mg/ml and 7.5 mg/ml Huaier for 24, 48 and 72 h in three cell lines.

Given that Huaier treatment alters β-catenin dynamics, resulting in decreased nuclear β-catenin levels, the expression levels of the downstream Wnt/β-catenin target genes were assessed by real-time PCR and western blot analysis. The transcription of *DIXDC1*and *LRP6* was significantly decreased in both SKOV3 and SKOV3.ip1 cells. *WNT5A* was repressed in the SKOV3 cells. Similarly, Huaier treatment can suppress the expression of DIXDC1 and WNT5A in Hey cells. (Figure 10A). Cyclin D1, an important Wnt signaling molecule that regulates cell cycle progression, was also reduced by Huaier treatment in three cell lines in a time- and dose-dependent manner (Figure 10B).

### Huaier inhibited human ovarian xenografts tumor growth in mice

**Figure 11:**
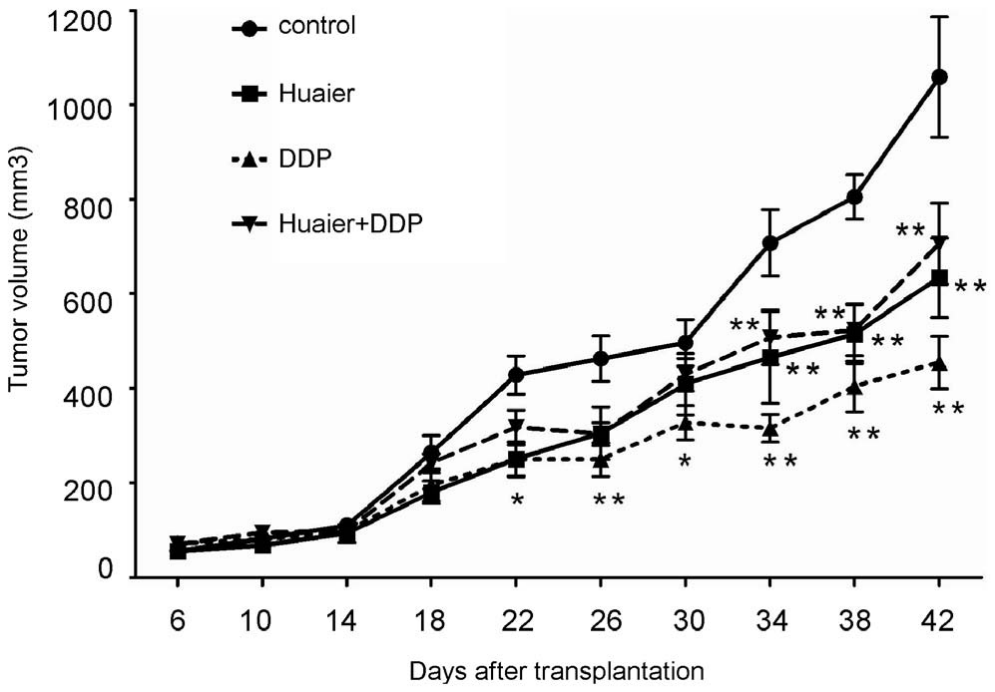
Huaier blocks xenograft tumor growth in vivo. SKOV3 cells (4 × 10^6^) were injected subcutaneously into female nude mice. The tumor sizes in the control and Huaier-, DDP- and Huaier + DDP-treated groups (6 mice in each group) were measured twice a week for up for 42 days after the tumor cell injection. The data are presented as means ± SD.* p<0.05 compared with the control.

To further investigate the effect of Huaier, we conducted xenograft experiments in mice. We found that Huaier treatment significantly inhibited the growth of SKOV3 cells compared with the control group but exerted no synergistic effect with cisplatin treatment (Figure 11).

## Discussion

Due to the low cure rate for advanced and recurrent ovarian cancers, many researchers are devoted to finding new treatment strategies. TCM, a rich source of new drugs, has aroused much interest. In addition to the anti-proliferation and pro-apoptotic effects found in other studies[6]–[8], we have provided evidence that Huaier can inhibit the invasion of ovarian cancer cells via the AKT/GSK3β/β-catenin pathway.

Huaier treatment had more evident cytotoxic and apoptotic effects in SKOV3.ip1 and Hey cells than in SKOV3 cells. These results indicated that SKOV3.ip1 and Hey cells, which have a higher metastatic potential, were more sensitive to the Huaier treatment. In agreement with this observation, the RPPA and western blot analyses revealed that phosphorylation of AKT at T308 was down-regulated in SKOV3.ip1 cells. AKT is an upstream activator of the mTOR pathway[14]. Ribosomal S6 kinase is a key target of mTOR, which controls protein synthesis in cells. Reduced S6 kinase activity leads to a decrease in protein synthesis and cell growth[15]. The reduction in the phosphorylation of S6 at S235–236 and S240–244 was also more apparent in the SKOV3.ip1 and Hey cells than in the SKOV3 cells. Our experiments indicated that Huaier exerts its anti-proliferative effects partly via the AKT/S6 kinase pathway, especially in ovarian cancer cells with a higher metastatic potential. We speculate that the differential effect may partially attribute to intrinsic gene profiles of cells with different metastatic potential. High- throughput methods such as mRNA microarray or protein array could help identify the specific profiles. More cell lines need to be tested to elucidate the phenomena in further study.

The invasion and migration assays showed that the invasive capability of ovarian cancer cells was significantly decreased after treatment with Huaier. As expected, β-catenin was one of several cell adhesion-associated proteins modulated by Huaier, as shown in the RPPA analysis and further confirmed by western blot analysis. β-Catenin is a component of the E-cadherin/cell–cell adhesion complex and is found predominantly in association with E-cadherin's cytoplasmic domain at cell-cell junctions[16]. Whereas the β-catenin present in adhesion complexes is thought to be relatively stable, upon the loss of E-cadherin function, liberated β-catenin is rapidly phosphorylated by GSK3β in the adenomatous polyposis coli (APC)/axin/GSK3β complex and subsequently degraded by the ubiquitin-proteasome pathway[17]. However, the loss of E-cadherin from the plasma membrane liberates β-catenin molecules, which may then migrate to the nucleus and associate with TCF/Lef transcription factors, thereby inducing genes that orchestrate invasion and migration[18].

In our study, treatment with Huaier resulted in a significant increase in the expression of total GSK3β and decrease in the phosphorylation of GSK3β at S9. We demonstrated that treatment of SKOV3, SKOV3.ip1 and Hey cells with Huaier decreased the cytosolic accumulation of β-catenin and inhibited the nuclear translocation of β-catenin. The decreased expression of β-catenin reflected its rapid degradation by proteolysis after its phosphorylation by GSK3β. In addition, knocking down GSK3β interfered with Huaier's down-regulation of β-catenin, which further indicates Huaier exerts it's role via GSK3β/β-catenin pathway. Many studies have shown that decreased E-cadherin expression enhances tumor cell invasion[19], [20]. In our study, the expression of E-cadherin significantly increased after Huaier treatment only in the Hey cells, but it did not change after Huaier treatment in the SKOV3 and SKOV3.ip1 cells, which might be incompatible with the reduced invasion capability observed in these cells. However, due to enhanced GSK3β expression, the dramatic reduction in both cytoplasmic and nuclear β-catenin may interfere with the expression of genes orchestrating invasion and migration.

Increased β-catenin nuclear signaling enhances the transcription of many genes that contribute to tumor progression[21], [22], including genes that modulate invasion and metastasis[23]. The detection of decreased Wnt target gene expression provides additional evidence in support of Wnt pathway inhibition. In this study, we demonstrated reductions in expression of *DIXDC1*, *LRP6* and *WNT5A* upon Huaier treatment. *DIXDC1*, the human homolog of Ccd1, is a positive Wnt signaling pathway protein that functions downstream of Wnt and upstream of axin and that targets p21 and cyclin D1 to promote colon cancer cell proliferation[24]. *LRP6* recruits axin and Dishevelled to the plasma membrane, thereby disrupting the degradation of β-catenin and facilitating β-catenin nuclear translocation[25]. *WNT5A* has been implicated in almost all aspects of non-canonical Wnt signaling. There is conflicting evidence as to whether *WNT5A* has a tumor-promoting or tumor-suppressing role. Emerging evidence suggests that the functions of *WNT5A* can be altered depending on the availability of key receptors[26]. Cyclin D1, a key Wnt target gene, acts as a mitogenic signal sensor. Cyclin-dependent kinases (CDKs) 4 and 6 are cyclin D1 binding partners. Activated cyclin D1/CDK4 and cyclin D1/CDK6 complexes facilitate the progression from G1 to S phase[27]. Our results are consistent with Burkhalter's finding that β-catenin plays a key role in epithelial ovarian cancer invasion and metastasis[28].

GSK3β has been reported to be very important in ovarian cancer. The mechanism by which Huaier strengthens GSK3β's expression still needs to be explored. The PI3K/AKT signaling cascade is one of the signaling pathways that lead to inhibition of GSK3β by increasing its phosphorylation at S9[29], [30]. GSK3β can also be phosphorylated by other proteins, including protein kinase C, p70 ribosomal S6 kinase, and p90RSK[31]; thus, multiple mechanisms have evolved to survey and control the activity of GSK3β. Our results showed that treatment with Huaier decreases the phosphorylation of AKT, suggesting that pAKT inhibition is related to the Huaier-induced increase in GSK3β expression. However, further investigation is required to elucidate whether other protein kinases are involved in this effect.

In summary, in addition to inhibiting cell growth by reducing proliferation and inducing apoptosis, Huaier reduces cell mobility in ovarian cancer cells via the AKT/GSK3β/β-catenin signaling pathway. Given its low toxicity, Huaier's effect on GSK3β/β-catenin signaling makes it an attractive way to target invasion in epithelial ovarian cancer cells. Further studies are needed to clarify additional mechanisms of Huaier's effects on ovarian cancer.

## Supporting Information

Figure S1 E-cadherin expression was not significantly changed in SKOV3 and SKOV3.ip1 cells, but increased in Hey cells by Huaier treatment, as measured by western blot (A) and immunocytochemistry (B).
(TIF)

Table S1 Proteins and phosphorylation sites used in RPPA studies.
(DOC)
