# Supplementary figures and images for: Huaier Aqueous Extract Inhibits Ovarian Cancer Cell Motility via the AKT/GSK3β/β-Catenin Pathway
Source: PLoS One. 2013 May 8;8(5):e63731. doi: 10.1371/journal.pone.0063731 (PMC3648479; doi:10.1371/journal.pone.0063731)

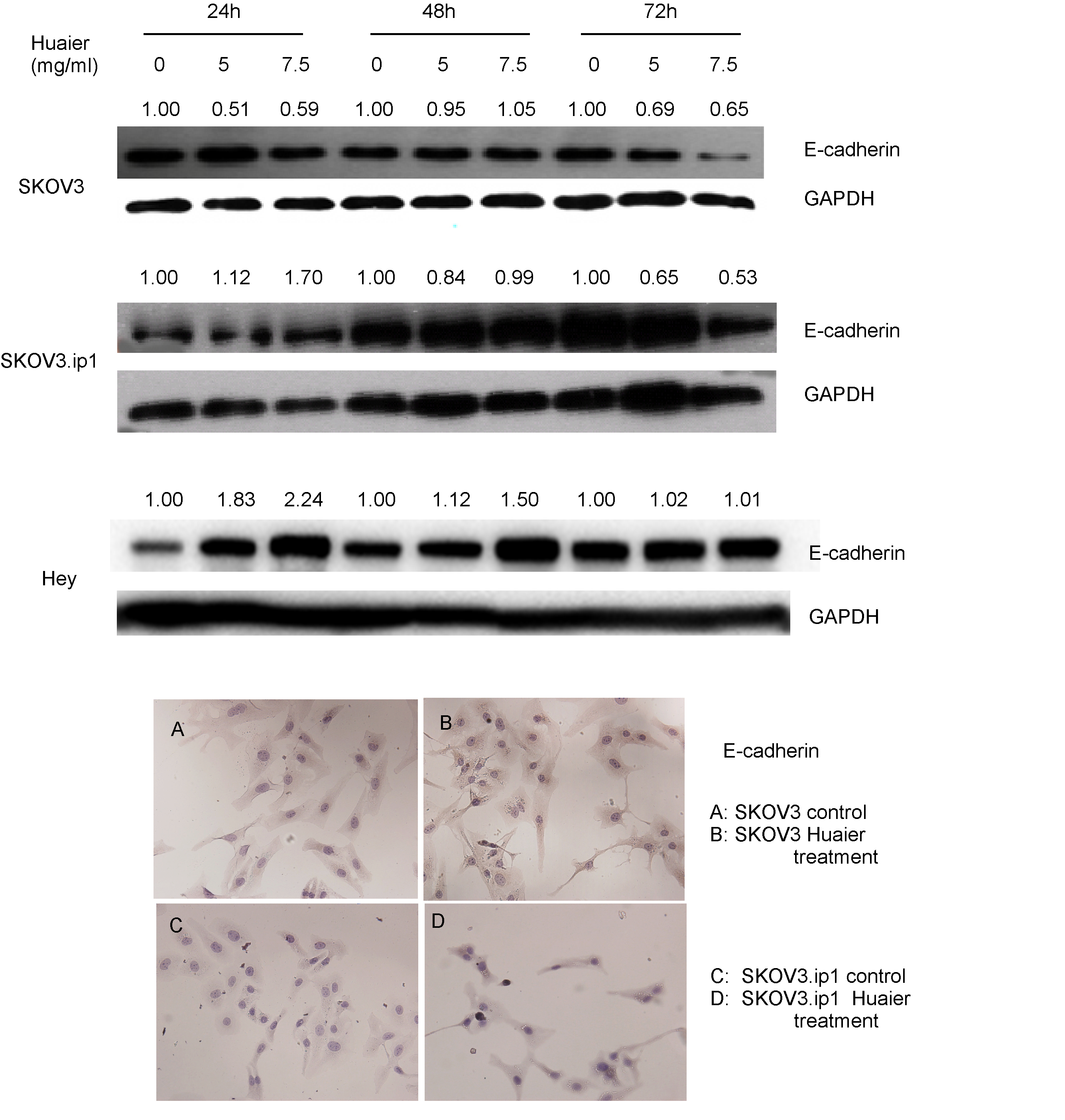

Supplement: Figure S1 — E-cadherin expression was not significantly changed in SKOV3 and SKOV3.ip1 cells, but increased in Hey cells by Huaier treatment, as measured by western blot (A) and immunocytochemistry (B). (TIF) [file pone.0063731.s001.tif]
