# Supplementary material for: Huaier Aqueous Extract Inhibits Ovarian Cancer Cell Motility via the AKT/GSK3β/β-Catenin Pathway
Source: PLoS One. 2013 May 8;8(5):e63731. doi: 10.1371/journal.pone.0063731 (PMC3648479; doi:10.1371/journal.pone.0063731)
Supplement: Table S1 — Proteins and phosphorylation sites used in RPPA studies. (DOC) [file pone.0063731.s002.doc]

Table S1. Proteins and phosphorylation sites used in RPPA studies

| ACC1 | Caspase3 | FKHRL/FoxO3 a | p27_pT198 | S6 |
| --- | --- | --- | --- | --- |
| 4EBP1/p65 | Caspase7 | FoxO3a/pS318 | p38/pT180 | S6/pS235-236 |
| 4EBP1/pT37 | Caveolin-1 | GATA3 | p38MAPK | S6/pS240-244 |
| 4EBP1/pT70 | CD31 | GSK3/pT21 | p53 | SMAD4 |
| 4EBP1 | CHK1/pS345 | GSK3b | p70S6K/pT389 | SMAD3 |
| ACC/pS79 | CHK1 | HER2/pY1248 | p70S6K | Snail |
| AIB1 | CHK2/pT68 | HER2 | p90RSK/pT359_S363 | SRC/pY416 |
| AKT/pS473 | CHK2 | HSP70 | PARP/cleaved | SRC/pY527 |
| AKT/pT308 | Collagen VI | IGFBP2 | PCNA | SRC |
| AKT | COX2 | IGF1Rb | PDK1/pS241 | STAT3/pY705 |
| AMPK/pT172 | CDC2 | INPP4B | Paxillin | STAT5 |
| AMPK | Cyclin B1 | IRS1 | PI3K/p110a | Stathmin |
| Annexin | Cyclin E1 | JNK2 | PKCa/pS657 | hSPARC |
| AR | CyclinD1 | KU80 | Pea15 | SPARC |
| ATM | Cyclin E2 | IKBa-pS32 | Pea15_pS116 | Tau |
| BaK | EGFR | H2AXg_pS139 | PKCa | Taz |
| BCL_X | EGFR­_pY992 | MAPK/pT202 | NF2(C) | Taz_pS79 |
| Bcl_xL | EGFR_pY117 | MEK1_pS217 | PI3K/p85 | Transglutaminase |
| Beclin | DJ1 | MEK1 | PR | Tuberine |
| Bid | DNA_PK | HIF1a | PTCH | VASP |
| Beta-Catenin | DNA_PKcs_pS2056 | p53_pS15 | Pras40_pT24 | VEGF |
| Bcl2 | DNA_PKcs_pT2609 | MSH2 | Raf_C_pS388 | VEGFR2 |
| BIM | DNA_PKcs_Y393 | MSH6 | NFKB_p65_pS536 | XRCC1 |
| cJUN/pS73 | eEF2 | K-ras | Raf_B | Y_box_BP1 |
| Ckit | eEF2K | MIG6 | Raf_C | YAP |
| Claudin7 | eIF4E | Mre11 | PTEN | YAP_pS127 |
| CD20 | ER | Notch1 | Rab25 | YB1_pS102 |
| CMyc | ERa/pS118 | Notch3 | RAD50 | X53BP1 |
| E-cadherin | FAK | P21 | RAD51 |  |
| N-cadherin | Fibronectin | P27 | Rb/pS807 |  |
| P-cadherin | Bax | p27_pT157 | Rb |  |
